# The Royal Australian Chemical Institute’s 10th National Convention

**DOI:** 10.1155/MBD.1995.123

**Published:** 1995


					The Royal Australian Chemical Institute's
10th National Convention
will be held at the
Adelaide Convention Centre,
South Australia
27 September- 2 October
1995
The Convention theme is
'Chemistry Serving Society'
Further information can be obtained from:
Hartley Management Group Pty Ltd
PO Box 2167 Kent Town SA 5071
Ph618 3612220 Fax6183640031
123

				

